# Quantification of Severe Acute Respiratory Syndrome Coronavirus 2 Binding Antibody Levels To Assess Infection and Vaccine-Induced Immunity Using WHO Standards

**DOI:** 10.1128/spectrum.03709-22

**Published:** 2023-01-23

**Authors:** Olivier Pernet, Steven Balog, Eric S. Kawaguchi, Chun Nok Lam, Patricia Anthony, Paul Simon, Rani Kotha, Neeraj Sood, Howard Hu, Andrea Kovacs

**Affiliations:** a Department of Pediatrics, Maternal, Child and Adolescent Center for Infectious Diseases and Virology, Keck School of Medicine, University of Southern California, Los Angeles, California, USA; b Department of Population and Public Health Sciences, University of Southern California, Los Angeles, California, USA; c Los Angeles County Department of Public Health, Los Angeles, California, USA; d Schaeffer Center for Health Policy & Economics, University of Southern California, Los Angeles, California, USA; e Sol Price School of Public Policy, University of Southern California, Los Angeles, California, USA; National Institute of Allergy and Infectious Diseases

**Keywords:** SARS-CoV-2, assay standardization, humoral immunity, hybrid immunity, immunization, serology

## Abstract

Severe acute respiratory syndrome coronavirus 2 (SARS-CoV-2) binding antibody (Ab) levels following vaccination or natural infection could be used as a surrogate for immune protection if results of serological assays were standardized to yield quantitative results using an international standard. Using a bead-based serological assay (Luminex xMAP), anti-receptor binding domain (anti-RBD) Ab levels were determined for 1,450 participants enrolled in the Los Angeles Pandemic Surveillance Cohort (LAPSC) study. For 123 participants, SARS-CoV-2 binding antibody unit (BAU) levels were also quantified using WHO standards and then compared to the semiquantitative results. Samples were chosen to represent the range of results and time from vaccination. Antibody levels and decay rates were then compared using unadjusted and adjusted linear regression models. The linear range of the assay used in this study was determined to be 300 to 5,000 mean fluorescence intensity units (MFI). Among the fully vaccinated groups (vaccinated only and vaccinated with past infection), 84.8% had anti-RBD MFI values above the linear range of >5,000 MFI, and 33.8% had values of >15,000 MFI. Among vaccinated participants with past infection (hybrid immunity), 97% had anti-RBD values of >5,000 MFI and 70% (120/171) had anti-RBD values of >15,000 MFI. In the subgroup quantified using the WHO control, BAU levels were significantly higher than the semiquantitative MFI results. In vaccinated participants, Ab decay levels were similar between infected and noninfected groups (*P* = 0.337). These results demonstrate that accurate quantitation is possible if standardized with an international standard. BAU can then be compared over time or between subjects and would be useful in clinical decision making.

**IMPORTANCE** Accurate quantification of SARS-CoV-2-specific antibodies can be achieved using a universal standard with sample dilution within the linear range. With hybrid immunity being now common, it is critical to use protocols adapted to high Ab levels to standardize serological results. We validated this approach with the Los Angeles Pandemic Surveillance Cohort by comparing the antibody decay rates in vaccinated participants and vaccinated infected participants.

## INTRODUCTION

**FIG 1:**
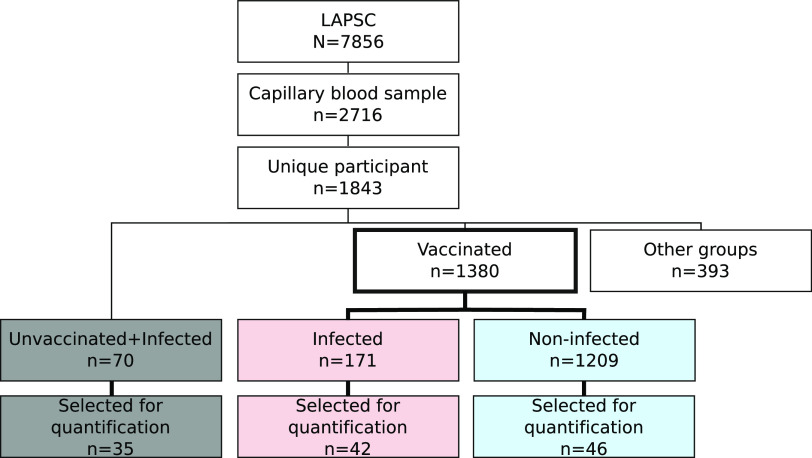
The Los Angeles Pandemic Surveillance Cohort (LAPSC). A total of 2,716 blood samples were collected from 1,843 participants of the cohort, of whom 1,450 could be classified as vaccinated with no history of past infection and no N antibody (*n* = 1209), vaccinated with previous infection (*n* = 171), and unvaccinated with previous infection (*n* = 70). Partially vaccinated participants were excluded from that study.

Now that we are in the third year of the severe acute respiratory syndrome coronavirus 2 (SARS-CoV-2) pandemic, it has become increasingly important to find surrogate markers of immune protection. Assessments of neutralizing antibody (Ab) and T cell responses following vaccination or natural infection are gold standards of immune protection (1, 2). However, these assays are not readily available or standardized (3–5). Total blood SARS-CoV-2 IgG antibodies are highly correlated with neutralizing antibody levels following vaccination and/or past infection. If properly standardized (6–8), IgG Abs could be used to make clinical decisions for treatment or prophylaxis in individual patients who may be at high risk for severe disease. There are over 80 SARS-CoV-2 FDA emergency use authorization (EUA)-approved antibody assays to assess total IgG Abs for SARS-CoV-2 (5, 9) and additional in-house protocols (1, 10). Most assays are semiquantitative and not standardized against a universal control (5). The World Health Organization (WHO) has developed standards using international binding antibody units (BAU), which can be used to assess quantitative antibody levels (2–4, 11). In this study, we determined Ab levels following vaccination and/or infection in a large cohort of participants enrolled in the Los Angeles Pandemic Surveillance Cohort (LAPSC) study (Fig. 1). In a selected subgroup, SARS-CoV-2 BAU were quantified using the WHO standards.

## RESULTS

### Demographic and clinical characteristics of study population.

The characteristics for the 1,450 participants characterized by vaccine and infection status were similar for those who were vaccinated only (*n* = 1,209), vaccinated and infected (*n* = 171), and infected only (*n* = 70) (Table 1). The majority were 30 to 64 years of age (75.3%, 74.3%, and 80%, respectively) and white or Hispanic (70%, 74.8%, and 74.3%, respectively), with women being slightly overrepresented (54.3%, 61.4%, 55.7%, respectively). Among the vaccinated participants (with or without infection), the majority received the BNT162b2 (40.4% and 50.4%) and MRNA-1273 vaccines (48.5% and 41.3%, respectively). Anti-receptor binding domain (anti-RBD) levels were different by cohort group, with the highest levels among those who were vaccinated with past infection. The selected comparison subset of 123 participants in the quantification substudy also had similar characteristics (Table 2).

**TABLE 1:** Demographics of study population (*n* = 1,450) for quantitative anti-RBD analysis

Characteristic	No. (%) for:			*P* value^*a*^
	Fully vaccinated and uninfected individuals (*n* = 1,209 [83.4%])	Fully vaccinated and infected individuals (*n* = 171 [11.8%])	Unvaccinated and infected individuals (*n* = 70 [4.8%])	
Age, yrs				0.008*
18–29	160 (13.2)	34 (19.9)	12 (17.1)	
30–39	588 (48.6)	79 (46.2)	42 (60.0)	
50–64	323 (26.7)	48 (28.1)	14 (20.0)	
≥65	138 (11.4)	10 (5.8)	2 (2.9)	
Race/ethnicity				<0.001
Hispanic	393 (32.5)	89 (52.0)	32 (45.7)	
White	453 (37.5)	39 (22.8)	20 (28.6)	
Black	79 (6.5)	13 (7.6)	9 (12.9)	
Asian	228 (18.9)	24 (14.0)	7 (10.0)	
Other	56 (4.6)	6 (3.5)	2 (2.9)	
Gender				0.28*
Male	545 (45.1)	64 (37.4)	31 (44.3)	
Female	657 (54.3)	105 (61.4)	39 (55.7)	
Nonbinary/transgender	7 (0.6)	2 (1.2)	0 (0.0)	
Income				0.001*
<$50,000	330 (27.3)	60 (35.1)	32 (45.7)	
$50,000–$99,999	396 (32.8)	54 (31.6)	18 (25.7)	
$≥100,000	413 (34.2)	46 (26.9)	12 (17.1)	
Prefer not to answer	70 (5.8)	11 (6.4)	8 (11.4)	
Vaccine type				0.03**
Ad26.COV2.S	93 (7.7)	19 (11.1)		
MRNA-1273	499 (41.3)	83 (48.5)		
BNT162b2	609 (50.4)	69 (40.4)		
Unsure	8 (0.7)	0 (0.0)		
Anti-RBD MFI level^*b*^				<0.001
≤5,000	204 (17)	5 (3)	33 (47)	
5,000–10,000	318 (26)	14 (8)	21 (30)	
10,000–15,000	340 (28)	32 (19)	9 (13)	
15,000–20,000	299 (25)	95 (56)	5 (7)	
>20,000	48 (4)	25 (15)	2 (3)	

a *, *P* value calculated via Fisher’s exact test using simulated *P* values from 10,000 Monte Carlo replicates; **, comparison of Ad26.COV2.S, MRNA-1273, and BNT162b2 between fully vaccinated participants that were infected and uninfected.

b For the 1,209 fully vaccinated and uninfected individuals, the median anti-RBD value was 11,443.00 (IQR, 6,698.00, 15,653.00); for the 171 fully vaccinated and infected individuals, the median was 17,263.50 (IQR, 14,023.50, 19,338.00); and for the 70 unvaccinated and infected individuals, the median was 5,946.50 (IQR, 2,614.50, 9,616.50).

**TABLE 2:** Demographics of selected subset (*n* = 123)

Characteristic	No. (%) for:			*P* value^*a*^
	Fully vaccinated and uninfected individuals (*n* = 46)	Fully vaccinated and infected individuals (*n* = 42)	Fully vaccinated and uninfected individuals (*n* = 35)	
Age, yrs				0.57*
18–29	8 (17.4)	11 (26.2)	6 (17.1)	
30–49	18 (39.1)	18 (42.9)	20 (57.1)	
50–64	16 (34.8)	10 (23.8)	8 (22.9)	
≥65	4 (8.7)	3 (7.1)	1 (2.9)	
Race/ethnicity				0.40*
Hispanic	19 (41.3)	27 (64.3)	21 (60.0)	
White	17 (37.0)	6 (14.3)	8 (22.9)	
Black	2 (4.3)	2 (4.8)	1 (2.9)	
Asian	7 (15.2)	6 (14.3)	4 (11.4)	
Other	1 (2.2)	1 (2.4)	1 (2.9)	
Gender				0.41*,***
Male	17 (37.0)	18 (42.9)	18 (51.4)	
Female	29 (63.0)	23 (54.8)	17 (48.6)	
Nonbinary/transgender	0 (0.0)	1 (2.4)	0 (0.0)	
Income				0.25*
<$50,000	13 (28.3)	14 (33.3)	17 (48.6)	
$50,000–$99,999	18 (39.1)	13 (31.0)	9 (25.7)	
$≥100,000	14 (30.4)	10 (23.8)	6 (17.1)	
Prefer not to answer	1 (2.2)	5 (11.9)	3 (8.6)	
Vaccine type				0.83*
MRNA-1273	22 (47.8)	22 (52.4)	0 (0.0)	
BNT162b2	24 (52.2)	20 (47.6)	0 (0.0)	

a *, *P* value calculated via Fisher’s exact test using simulated *P* values from 10,000 Monte Carlo replicates; ***, comparison only of males to females across the different subgroups.

### Anti-RBD antibody levels.

Among the 1,380 unique samples from the fully vaccinated groups (vaccinated only and vaccinated with past infection), 84.8% (1,171/1,380) had anti-RBD values of >5,000 mean fluorescence intensity units (MFI), and 33.8% (467/1,380) had values of >15,000 MFI. Among the 171 fully vaccinated participants with documented past infection, 97% (166/171) had anti-RBD values of >5,000 MFI and 70% (120/171) had anti-RBD values of >15,000 MFI. Finally, among the 70 unvaccinated and previously infected participants, only 52.9% (37/70) had anti-RBD values of >5,000 MFI and 10% (7/70) had anti-RBD values of >15,000 (Table 1).

### Using the WHO standard to establish the linear range of the assay.

**FIG 2:**
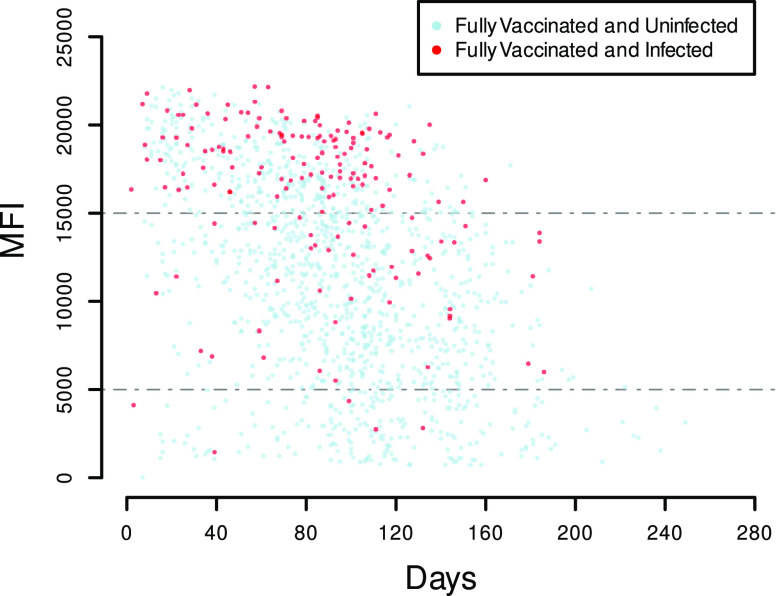
Anti-RBD levels in the fully vaccinated population. Blood samples from unique participants were analyzed using the Luminex xMAP SARS-CoV-2 multiantigen antibody assay protocol. A total of 84.8% of the samples had anti-RBD values of >5,000 MFI.

**FIG 3:**
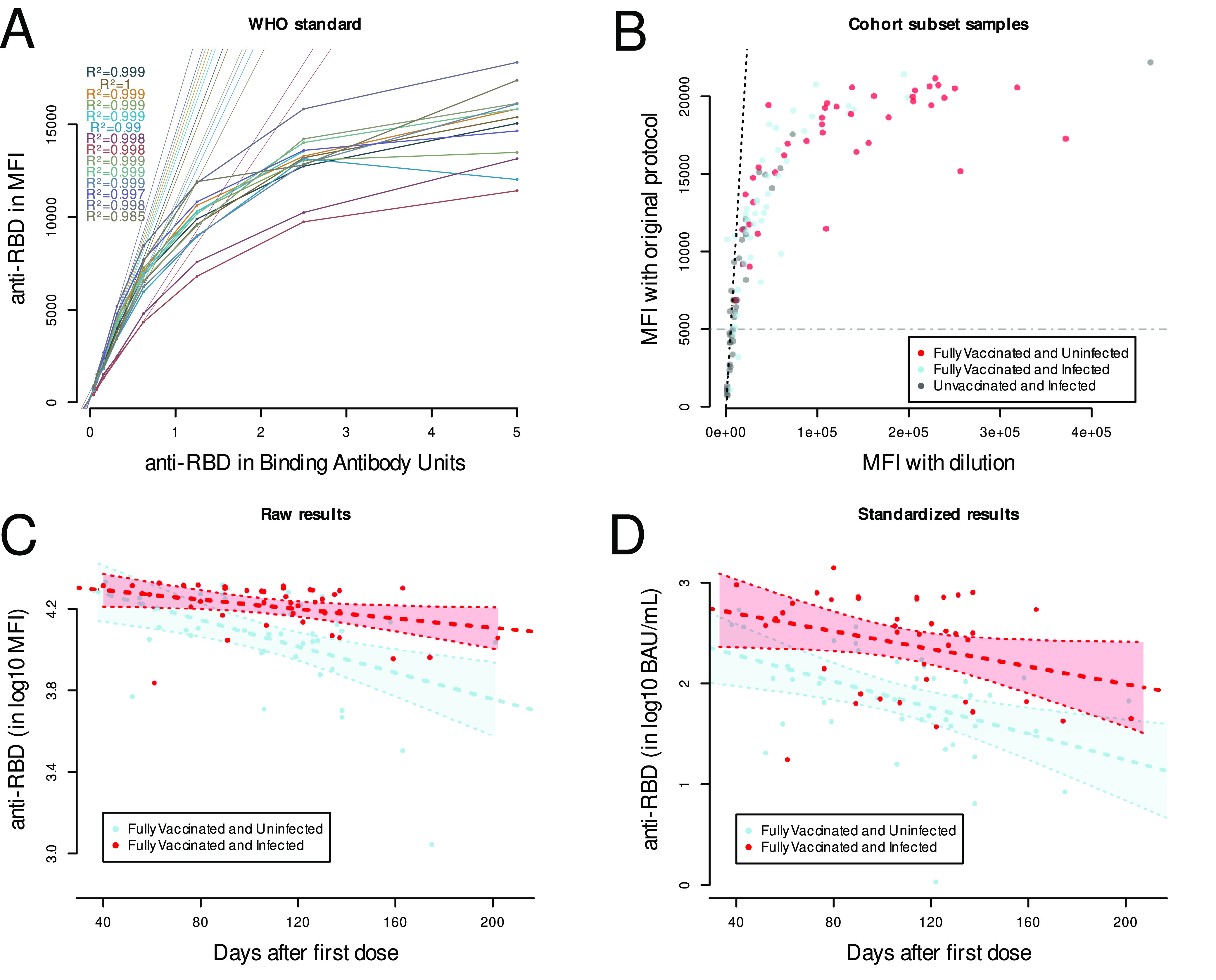
(A) Standard scale using the WHO standard. For each run, the WHO standard (EN63QG—20/136) was used to create a control scale with 2-fold serial dilutions from 5 binding antibody units (BAU) to 0.04 BAU per reaction. Serial dilutions were analyzed using the Luminex xMAP SARS-CoV-2 multiantigen antibody assay protocol, and a linear regression model was built for each of the curves using values of ≤5,000 MFI. (B) Dilution of serum samples. The serum samples were diluted to match the MFI of the linear range of the assay. The MFI were then adjusted by the dilution factor and plotted against the undiluted MFI. The 1:1 ratio line (black broken line) highlights the underestimation of undiluted samples for MFI values above 5,000. (C) Undiluted nonquantified MFI values were plotted against days since vaccination and a linear model was built to compare the vaccinated-only group (blue) and the vaccinated infected group (red). (D) Quantitative serology using the WHO standard. The linear regression models obtained in panel A and the diluted MFI from panel B were then used to accurately calculate the serum antibody levels in BAU corresponding to each MFI value. Values in BAU were then plotted against time since full vaccination as defined in Materials and Methods.

As shown in Fig. 2, the assay reached a technical ceiling, with maximum fluorescence intensities being measured between 20,000 and 25,000 MFI due to bead saturation. For this reason, we next assessed the linear range of the assay. A total of 14 replicates of 2-fold dilutions ranging from 5 BAU to 0.04 BAU were run to establish BAU/MFI correspondence across the range of the assay for quantitation, along with negative controls (Fig. 3A). For each of the 14 replicates, the coefficients of determination, *R*^2^, were established including either the points below 5,000 MFI only or the complete series. The coefficient of determination for the 14 corresponding linear regressions using only the points below 5,000 MFI was, on average, 0.9971 (standard deviation [SD] = 0.004), while the coefficient of determination including the points above 5,000 MFI was 0.8193 (SD = 0.057) (Fig. 3A).

### Quantification of SARS-CoV-2 Abs.

To assess the relationship between anti-RBD Ab using the semiquantitative protocol and our assay using the WHO control, we chose a subset of 123 participants as described in Materials and Methods. To be within the linear range, samples were diluted until their MFI results were ≤5,000. Of these, 21 samples (17.1%) required 10-fold dilution, 24 (19.5%) required 20-fold dilution, 29 (23.6%) required 40-fold dilution, 48 (39%) required 80-fold dilution, and 1 (0.8%) was diluted 100-fold. Those with past infection and vaccination required the highest dilution factor, while those with only past infection required fewer dilutions. The results confirmed that samples with anti-RBD values of ≤5,000 MFI were within the linear range of the assay and had a similar MFI (adjusted for dilution) in the optimized protocol. The results also confirmed that samples with anti-RBD values of >5,000 with the default protocol had significantly higher MFI values (adjusted for dilution) in the optimized protocol (Fig. 3B).

### Comparison of antibody decay rate using semiquantitative versus quantitative protocols.

Decay rates obtained using the semiquantitative (Fig. 3C) assay were compared to data obtained after dilution and quantification (Fig. 3D). Unadjusted analyses using data obtained by the semiquantitative protocol suggest that the decay rates were different between the fully vaccinated infected and fully vaccinated noninfected groups (Table 3, model A; *P* = 0.026). Critically, this relationship was not found when using the data obtained after standardization with the WHO control, indicating similar decay rates independent of infection status (Table 3, model B; *P* = 0.337). Since Fig. 3D and our unadjusted analysis suggest that there is no interaction between infection and weeks since vaccination (on log_10_ RBD using diluted data), we did not include an interaction in our adjusted model. Adjusted analyses (Table 3, model C) using the data obtained after standardization revealed that those who were infected and vaccinated had higher anti-RBD Ab than those who were vaccinated but never infected (*P* value < 0.001). Furthermore, antibody levels declined over time on the population level (*P* value < 0.001).

**TABLE 3:** Coefficient estimates, 95% confidence intervals, and *P* values from the unadjusted and adjusted linear regression analyses (*n* = 87)^*a*^

Model and parameter	Estimate	95% confidence interval	*P* value
Model A			
Prior infection	0.056	−0.14, 0.25	0.566
Wks since fully vaccinated	−0.008	−0.02, 0.00	0.090
Interaction	−0.015	−0.03, 0.00	0.026
Model B			
Prior infection	−0.300	−0.83, 0.23	0.262
Wks since fully vaccinated	−0.035	−0.06, −0.01	0.011
Interaction	−0.018	−0.05, 0.02	0.337
Model C			
Prior infection	−0.537	−0.737, −0.338	<0.001
Wks since fully vaccinated	−0.044	−0.062, −0.025	<0.001
Age, yrs			
18–29^*b*^			
30–49	−0.008	−0.291, 0.276	0.958
50–64	−0.013	−0.316, 0.289	0.931
65+	−0.079	−0.570, 0.412	0.751
Race/ethnicity			
Hispanic^*b*^			
White	−0.249	−0.522, 0.025	0.074
Other^*c*^	−0.111	−0.395, 0.172	0.437
Gender			
Male^*b*^			
Female	−0.60	−0.160, 0.280	0.587
Study wave			
Wave 1^*b*^			
Wave 2	−0.011	−0.328, 0.306	0.947
Model D^*d*^			
Prior infection	−0.595	−0.83, −0.36	<0.001
Wks since fully vaccinated	−0.044	−0.07, −0.02	0.002

a Model A, regresses prior infection status, weeks since fully vaccinated, and the corresponding interaction on undiluted nonquantified MFI values of RBD (on the log_10_ scale); model B, regresses prior infection status, weeks since fully vaccinated, and the corresponding interaction on diluted and quantified MFI values of RBD (on the log_10_ scale); model C, regresses prior infection status and weeks since fully vaccinated on diluted and quantified MFI values of RBD (on the log_10_ scale) adjusted for age, race/ethnicity, gender, and study wave; model D, sensitivity analysis using the same model as model C but removing the 13 individuals who either had no date of testing positive or tested positive after their most recent vaccination date (*n* = 74).

b Reference group.

c Black, Asian, and other subjects were combined into one category due to small cell counts.

d Estimates were omitted from adjustment covariates for brevity.

Finally, among the 42 individuals in the infected and vaccinated group, 13 either reported no date of testing positive or tested positive after their most recent vaccination date. A sensitivity analysis removing these 13 individuals was performed, and the results were consistent with what we observed previously (Table 3, model D).

## DISCUSSION

This is one of the largest studies to date to comprehensively evaluate SARS-CoV-2 Abs in a representative population of Los Angeles County (LAC). Our study had several notable findings. First, we were able to quantify SARS-CoV-2 anti-RBD BAU levels using the WHO standard. Second, the majority of the samples reached the ceiling of the semiquantitative assay and did not accurately depict quantitative antibody level variations, especially for participants recently vaccinated and those with hybrid immunity. Third, we found that those who were infected and then vaccinated had higher antibody levels than those only vaccinated. Finally, we show that the antibody kinetics and decay rates of anti-RBD levels were the same for those vaccinated only and those vaccinated with previous infection once results were standardized to the linear range of the WHO control and adjusted to time from vaccination. This study highlights the importance of standardizing serological results in order for them to be useful for clinical decision making and long-term cohort follow-up, especially in the context of SARS-CoV-2 hybrid immunity.

There are over 80 serological assays currently listed by the FDA (5, 9). Aside from widely used assays from major biotech companies such as Elecsys (Roche Diagnostics International Ltd.) (12, 13), xMAP SARS-CoV-2 (Luminex) (14), and Abbott-N (13, 15–17), in-house assays remain common (1, 10). Most report semiquantitative results with no international standard adjustments and use different output formats (5): arbitrary units (AU) per milliliter (5, 12, 13, 15–17), optical density (1, 10, 18), MFI (14), or percentage of participants above a threshold (14). Different assays might also have different dynamic and linear ranges (5, 15, 16). Unfortunately, for SARS-CoV-2 assays, the dynamic and linear ranges are rarely provided (15, 16), and antibody levels in samples with saturating values might be underestimated, as we observed for most of the participants from this study, especially those both infected and vaccinated.

Performance studies have shown disparities between assays: for example, the Abbott SARS-CoV-2 IgG II assay is linear up to 38,365 AU/mL (15). Recently, another study using the same assay showed titers above 80,000 AU/mL in boosted participants (17). In another study, at least 5 recently infected participants tested with another assay using a different unit system (Roche) reached 2,500 AU/mL, which suggest a ceiling effect and therefore a potential underestimation of their actual antibody levels (12). We can expect more people to reach saturating levels of Ab due to the increase in booster dose uptake and breakthrough and repeat infections (17). Assays that were designed in the early days of the outbreak might not have the range for current and future BAU levels, especially in the case of hybrid immunity. The impact might be a general underestimation of anti-SARS-CoV-2 antibody levels in long-term serological studies. The standard serum developed by the WHO has now been replicated by other major agencies, including the U.S. National Cancer Institute (3). Systematically using standard sera with identical units will compensate for the variation between protocol, machine, location, and operator and, importantly, between assays (2).

Anti-RBD Abs are often used as proxy for neutralizing Abs, as they have been shown to be correlated (6–8). Moreover, some assays rely specifically on serum neutralization (1, 19–21). Although this approach allows for providing results as universal 50% and 90% tissue culture infective doses (TCID_50_ and TCID_90_), it misses a whole aspect of the humoral response by ignoring nonneutralizing Abs (5). This can be a critical issue, as most of the antibody response following SARS-CoV-2 vaccination with mRNA vaccine is nonneutralizing (22), and nonneutralizing Abs have been shown to contribute to other aspects of the immune system activity, such as phagocytosis by monocytes and antibody-dependent cellular cytotoxicity (ADCC) (23). More generally, the production and maintenance of Abs require a complete immune system. For example, functioning humoral immunity is dependent on helper T cell and antigen-presenting cells. Thus, quantification of SARS-CoV-2-specific Abs can be used not only to assess the humoral response but also, to some extent, to evaluate some aspect of the cellular one.

In this study, we used the Luminex assay, as it has the ability to measure simultaneously SARS-CoV-2 anti-nucleocapsid (anti-N), anti-RBD, and anti-spike (anti-S) antibodies to identify participants with previous infection and to evaluate vaccine response. Furthermore, by adding the WHO standards, we achieved accurate quantification. There are also many checks on the system. In addition to positive- and negative-control samples, the assay uses control beads that measure total IgG levels and ratio between IgA and IgM. This allows for the detection of false-negative results and immune conditions that may interfere with humoral response, for example, immunosuppressive treatment and infections.

Despite using a bead-based protocol having a greater antigen-displaying surface than enzyme-linked immunosorbent assay (ELISA), the anti-RBD levels were so high that antigen saturation was reached, thus the need to dilute the samples and quantitate results using an external standard such as the WHO standard. Diluting the samples revealed the actual difference between groups and the actual decay trends of the anti-RBD. Importantly, the dilution factor had to be adjusted to each sample to match the linear range of the assay. Given that ELISA-based assays also would have a saturation point, our study results would apply for other assays that use a similar format. As we have shown with this study, most samples needed to be diluted in order to be accurately quantified. Indeed, this specific assay (like many others) was designed before vaccination was available and aimed to detect past infection. With vaccination, boosters, and hybrid immunity now common, higher antibody titers are now routinely found.

There are some limitations to this study. It was performed from spring-summer 2021, before the rise of the most recent variants, such as Delta and Omicron. This is especially relevant since the Omicron variants (from BA1 to now BA4/5) accumulate a significantly higher number of mutations in the spike than their predecessors (20, 21, 24, 25). (As epitopes vary, it is difficult to predict how these mutations will impact hybrid immunity and antibody kinetics, as recently shown by Reynolds et al. [26, 27]). Another difference between 2022 and 2021 is that hybrid immunity in the first part of 2021 (described in this paper) is mostly a product of vaccination after infection. In late 2021 and 2022, breakthrough infections became more common due to the rise of the Delta and Omicron variants (19–21, 24, 25), especially in Los Angeles, where the majority of adults are vaccinated (28). Breakthrough infections can occur in vaccine nonresponders or can happen in a successfully vaccinated participant whose neutralizing immunity has waned over time (29) or if the variants escape immunity (20, 24, 25, 30–32).

In summary, using a universal standard such as the WHO control, we demonstrated that quantitative Ab levels can be obtained and reported in BAU. Diluting samples to be within the linear range of the WHO controls allowed us to quantify antibody levels. Dilution was necessary to get accurate results due to the extremely high Ab levels following vaccination or combined infection and vaccination. In quantified samples, we confirmed that anti-RBD antibody levels decrease over time after vaccination/and or infection. Importantly, we observed similar decay rates among the vaccinated participants and those with past infection and vaccination. With hybrid immunity being now common since the emergence of the Delta and Omicron variants, it is critical to use protocols adapted to high Ab levels to standardize serological studies.

## MATERIALS AND METHODS

### Study population.

The Los Angeles Pandemic Surveillance Cohort Study, a collaboration between University of Southern California (USC) and the Los Angeles County Department of Public Health (LACDPH), enrolled 7,856 adult participants representative of the Los Angeles County (LAC) population in two waves between 9 April and 25 July 2021 as previously described (14, 33). A subgroup of 1,843 participants provided capillary serum samples collected using a Tap-II serum collection device (Seventh Sense Biosystems, Boston, MA, USA). The study was approved by the LACDPH and USC institutional review boards (IRB), and electronic informed consent was obtained from each participant. LRW (a Material company) implemented the recruitment process using its online member platform. Participants responded to a comprehensive questionnaire available in English and Spanish that included demographic, clinical, and epidemiologic data and history of vaccination and past infection. Survey items used in the study analysis have been described by Nicholas et al. (34). This study followed the Strengthening the Reporting of Observational Studies in Epidemiology (STROBE) reporting guideline (35).

### Serology.

We used a semiquantitative bead-based assay to determine anti-RBD, anti-S, and anti-N antibody levels according to the manufacturer’s protocol (xMAP SARS-CoV-2 multiantigen antibody assay; Luminex, Austin, TX, USA). Briefly, 10 μL of participant serum was diluted 1:400 in wash buffer. Fifty microliters of Luminex microsphere beads was added to 50-μL diluted samples and incubated for 60 min with agitation. Samples were then washed using a BioTek 50TS plate washer (Biotek, Winooski, VT, USA). Detection reagent was added for 60 min before a final wash. Mean fluorescence intensity was measured using a Luminex MAGPIX and xPonent software v.4 (Luminex). Values for RBD, S1, and N Abs are reported separately, and levels of <300 mean fluorescence intensity units (MFI) are negative, while values of ≥700 MFI are positive. The assay has a dynamic range of 0 to 25,000 MFI. The assay was validated using negative and positive external controls from Luminex (from the Luminex xMAP SARS-CoV-2 multiantigen antibody EUA assay kit; Luminex) and Bio-Rad (Bio-Plex Pro human IgG SARS-CoV-2 positive and negative controls 12014774 [reference number 390600 for positive and 390300 for negative; Bio-Rad, Hercules, CA, USA]). The threshold for positivity provided by the manufacturer was confirmed using pre-2019 samples. Past infection is reported with a positive result for anti-N Ab of ≥700 MFI along with a value for either anti-S1 or anti-RBD of ≥700 MFI. For this study, we focused on RBD antibody levels.

### Study definitions.

History of vaccine dates, self-reported positive quantitative PCR (qPCR)/antigen test dates, and antibody levels (anti-RBD, anti-S, and anti-N levels) were used to classify participants. A total of 2,716 blood samples were collected from 1,843 participants of the cohort; of these participants, 1,450 could be classified as (i) fully vaccinated with no history of past infection and anti-N Ab of ≤700 MFI (*n* = 1,209), (ii) fully vaccinated with previous infection with anti-N Ab (*n* = 171), and (iii) unvaccinated with previous infection (*n* = 70). All others (partially vaccinated, etc.) were excluded (Fig. 1). Participants with two visits were only assessed at the visit where they were classified into these groups. If two visits matched the criteria, only the second visit data were kept. Participants were defined as fully vaccinated if they received two doses of the MRNA-1273 or BNT162b2 vaccine and were ≥14 days after the second dose or received one dose of the Ad26.COV2 vaccine and were ≥14 days after vaccination date.

### Quantitative serology.

To establish the linear range for quantification of the anti-RBD, 2-fold serial dilutions of the WHO standard positive control (WHO IgG standard EN63QG—20/136) were run to have a range of 5 BAU to 0.04 BAU per reaction (specifically, 5, 2.5, 1.25, 0.625, 0.3125, 0.15625, 0.078125, and 0.390625 BAU per reaction).

To quantify anti-RBD BAU using the WHO control, a subset of 123 participants (Fig. 1) were selected to represent three of the cohort subgroups as follows: (i) vaccinated only (*n* = 46), (ii) infected only (*n* = 35), and (iii) vaccinated and infected (*n* = 42) (Table 2). Five infected and fully vaccinated individuals were selected for each decile of days from vaccination. One uninfected and fully vaccinated individual was matched to each infected and fully vaccinated individual based on days since being fully vaccinated. Thus, for each 50 uninfected and fully vaccinated individuals, 50 infected and fully vaccinated individuals with a similar number of days from full vaccination (≥14 days) were chosen; 40 unvaccinated and infected individuals were also resampled.

To quantify anti-RBD levels, we used WHO controls in a subset of participants. Selected samples were diluted 10-, 20-, 40-, or 80- and 100-fold so that the respective MFI matched the linear range of the assay (300 to 5,000 MFI). In the same run, serial dilutions of samples were performed. Finally, a linear regression model on the WHO standard curve within the linear range was used to correlate the MFI values observed in diluted samples to the actual BAU value.

### Statistical analyses.

All analyses were conducted using R (version 4.0.5; R Foundation for Statistical Computing, Vienna, Austria). All statistical tests were two sided, and *P* values of <0.05 were considered statistically significant. Baseline demographic characteristics of our study population are presented as median with interquartile range (IQR) or frequency (percentage). Comparisons between continuous variables was performed using the Kruskal-Wallis test. Pearson’s chi-squared test or Fisher’s exact test, depending on cell counts, was used to compare categorical variables. An initial linear regression model was performed to assess the impact of infection and weeks since being characterized as fully vaccinated on anti-RBD levels (on the log_10_ scale) using the semiquantitative and diluted data. In addition, an interaction between infection and weeks since being characterized as fully vaccinated was also included to test for and model infection status-specific decay rates. A linear regression model adjusting for gender, age, race/ethnicity, and study wave was used to assess the impact of infection and weeks since being characterized as fully vaccinated on diluted anti-RBD levels (on the log_10_ scale).
